# SiCRNN: A Siamese Approach for Sleep Apnea Identification via Tracheal Microphone Signals

**DOI:** 10.3390/s24237782

**Published:** 2024-12-05

**Authors:** Davide Lillini, Carlo Aironi, Lucia Migliorelli, Leonardo Gabrielli, Stefano Squartini

**Affiliations:** Department of Information Engineering, Università Politecnica delle Marche, Via Brecce Bianche 12, 60131 Ancona, Italy; c.aironi@univpm.it (C.A.); l.migliorelli@univpm.it (L.M.); l.gabrielli@univpm.it (L.G.); s.squartini@univpm.it (S.S.)

**Keywords:** sleep apnea syndrome, deep learning, clinical decision support system, sleep apnea, OSA detection

## Abstract

Sleep apnea syndrome (SAS) affects about 3–7% of the global population, but is often undiagnosed. It involves pauses in breathing during sleep, for at least 10 s, due to partial or total airway blockage. The current gold standard for diagnosing SAS is polysomnography (PSG), an intrusive procedure that depends on subjective assessment by expert clinicians. To address the limitations of PSG, we propose a decision support system, which uses a tracheal microphone for data collection and a deep learning (DL) approach—namely SiCRNN—to detect apnea events during overnight sleep recordings. Our proposed SiCRNN processes Mel spectrograms using a Siamese approach, integrating a convolutional neural network (CNN) backbone and a bidirectional gated recurrent unit (GRU). The final detection of *apnea* events is performed using an unsupervised clustering algorithm, specifically *k-means*. Multiple experimental runs were carried out to determine the optimal network configuration and the most suitable type and frequency range for the input data. Tests with data from eight patients showed that our method can achieve a Recall score of up to 95% for *apnea* events. We also compared the proposed approach to a fully convolutional baseline, recently introduced in the literature, highlighting the effectiveness of the Siamese training paradigm in improving the identification of SAS.

## 1. Introduction

Sleep apnea syndrome (SAS) is a common but often undiagnosed condition, affecting around 2–4% of middle-aged men and 1–2% of women in developed countries [1]. SAS is characterized by a pause in breathing, which can last 10 s or more and persists despite the patient’s continued efforts to inhale [2,3]. This disorder leads to repeated blockages of the upper airways during sleep [4], causing reduced oxygen levels and frequent awakenings [5]. Lack of sleep contributes to daytime fatigue [6], increased risk of cardiovascular disease, and long-term cognitive impairment [7,8]. Therefore, early detection of SAS is crucial to reduce these associated risks. Currently, clinical evaluations are often based on clinician-generated ratings [9], which are sometimes derived from questionnaires administered to patients. However, this method is qualitative and depends heavily on the experience of the examiner. To overcome these limitations, instrumental tests such as polysomnography (PSG) are used to support diagnosis [10,11]. PSG is performed in a hospital setting, where patients are equipped with several sensors [12], which may include an elastic chest band, a finger pulse oximeter, a nasal cannula, and a position sensor to monitor and measure changes in the sleep–wake cycle [13,14]. The intrusive nature of PSG, requiring an overnight hospital stay, has led researchers to develop alternative systems of diagnostic support systems that can extend assessment to home environments [15]. These systems often use microphones for data collection [16], and the acquired data are then analyzed through machine learning (ML) or deep learning (DL) algorithms [17].

In [18], the focus is on analyzing tracheal and ambient microphone recordings to calculate breathing probabilities in short audio segments, using a voice activity detection (VAD) algorithm. However, when detecting *apnea* in recordings made under suboptimal conditions, statistical methods may perform less well than DL techniques.

To overcome these limitations, other proposed approaches for detecting SAS use DL methods with hand-crafted features, or end-to-end architectures that detect snoring sounds associated with SAS or the SAS events. Li et al. [19] implemented spectral subtraction to reduce acoustic noise, followed by an unsupervised method to segment episodes of snoring. The audio signals are then represented as visual geometry maps, which are subsequently fed into a convolutional neural network (CNN) for snoring-event classification. Similarly, Cheng et al. [20] proposed a two-phase snoring classification system. In the first phase, snoring-related features are extracted using techniques such as Mel-frequency cepstral coefficients (MFCC), filter banks, short-time energy, and linear predictive coding (LPC). These features are then used as inputs to a long-short-term memory (LSTM) network for the classification of snoring events. In [21], the authors proposed a CNN-based method to detect snoring sounds for diagnosing obstructive sleep apnea syndrome (OSA). It combines spectrogram, Mel spectrogram, and continuous wavelet transform (CWT) into a multi-channel input framework. The work proposed in [22] depicts a method for the real-time detection of OSAs using breathing sounds recorded during sleep, even in noisy environments. The input consists of audio recordings from a smartphone and signals from a PSG device, with noise added to simulate home environments. The method involves transforming breathing sounds into Mel spectrograms and using a deep neural network to classify *apnea* events. Saini et al. [23] introduced an approach for detecting OSAs and assessing their severity in adults using audio signal analysis. This method involves processing recorded audio samples, applying noise reduction, and performing analysis in both the time and frequency domains. Features are extracted using fast Fourier transform (FFT) and discrete wavelet transform (DWT), which are then used to train an artificial neural network (ANN).

In [24], the authors presented a method for detecting and classifying OSA events through audio spectrogram analysis. Their approach involves extracting Mel spectrograms from ambient audio recordings, which are then processed using a machine learning model that combines a pre-trained CNN for audio classification with a bidirectional long short-term memory (Bi-LSTM) network. Ding et al. [25] proposed an end-to-end approach by examining a subset of the dataset from Korompili et al. [26]. Their method uses Mel spectrograms feeding a VGG19 network for feature extraction, followed by an LSTM network to classify normal and apnea-related snoring events.

Most of these studies rely on proprietary datasets, which hinders the reproducibility of the proposed analysis. The only publicly available dataset is the one collected by Korompili et al. [26], which includes tracheal audio recordings with an average duration of five hours, collected from 193 patients diagnosed with SAS.

Building on the state-of-the-art approaches outlined above, the present study introduces an innovative framework that exploits a Siamese convolutional recurrent neural network (SiCRNN) for feature extraction, followed by a k-means clustering stage, to classify *apnea* and *non-apnea* events. We conducted an in-depth hyperparameter tuning analysis to identify the optimal configuration that balances accuracy and computational complexity, which is crucial for deploying our decision-support system in clinical settings with limited computational resources.

## 2. Materials and Methods

### 2.1. Dataset

To train, evaluate, and test the proposed DNN-based model, we gathered audio recordings of 40 patients, from a larger collection released by Korompili et al. [26]. The dataset includes a label for each apnea episode, stored in a separate metadata file, which marks its onset and offset times with a resolution of 500 ms. The labels were generated by combining analyses performed by expert clinicians and PSG responses.

The audio recordings in the collection were captured using the Clockaudio CTH100 device. It is a tracheal microphone, secured to the patient’s throat with an elastic band and designed to reduce wearing discomfort. The audio signals were originally recorded at 48,000 samples per second, to conform with the joint use of an additional ambient microphone [26]. However, in our study, we only considered recordings from the tracheal device, which operates within a narrow frequency band. Therefore, we downsampled the signals to 16 kHz, as this does not imply a loss of information. We then segmented the stream into contiguous windows of 6 s each, with no overlap, and established a criterion to assign a label to each of them: a window was labeled as *positive* if an apnea event extended throughout the entire duration, otherwise, it was labeled as *negative*, indicating the absence of apnea. A 32-band Mel spectrogram was calculated for each time window [27] and patient; the spectrograms were saved in two different folders according to the labels (i.e., *apnea* or *non-apnea*). Variants with fewer Mel bands, i.e., 25, 18, 12, and 8, were considered with the purpose of simulating the downsampling of the signal to, respectively, 8 kHz, 4 kHz, 2 kHz and 1 kHz. The STFT spectrogram without frequency scaling was also included at the lowest resolution.

This study faced computational constraints due to the high volume of audio data (1 TB, or 3.5 million seconds) in the dataset by Korompili et al. [26], which originally included 135,568 apnea event windows and 446,431 non-apnea event windows. Training a Siamese neural network exacerbated the challenge of handling large data volumes because it required generating all possible audio pairs for training, leading to a combinatorial explosion of over 110 billion combinations if the full dataset was used. To address this, we opted to use a subset of 40 patients, amounting to 30,000 instances. This reduced the input pairs to 450 million, making the training process computationally feasible while still maintaining sufficient data diversity and variability to ensure robust and effective model training.

**Figure 1:**
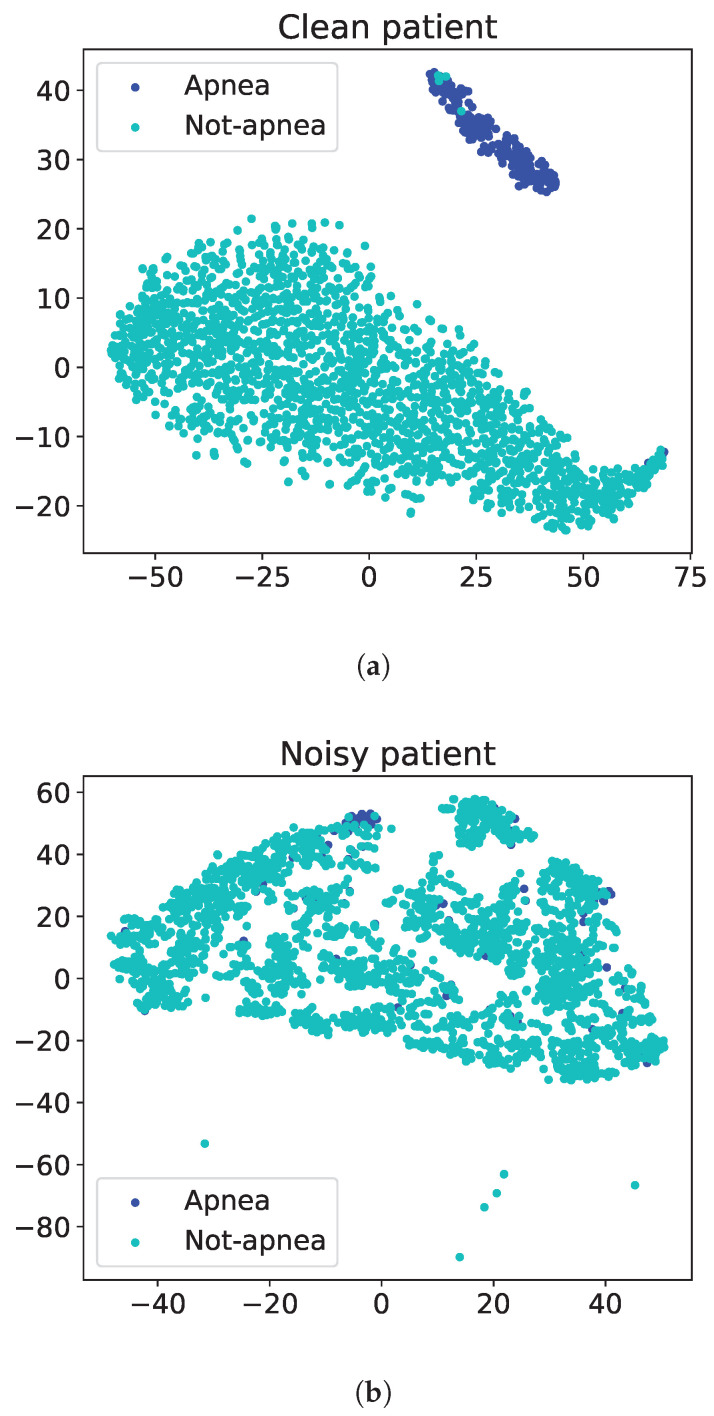
The scatter plots illustrate the output of the principal component (PCA) applied to the output of the final GRU layer in the SiCRNN model. The resulting embeddings are derived from two patients under two conditions: (**a**) noise-free patient embeddings and (**b**) noisy patient embeddings. The observed distances between the *apnea* and *non-apnea* clusters are 2.0 in the noise-free scenario and 0.87 in the presence of noise, respectively.

We conducted a preliminary analysis of the data from 40 patients to identify excessively noisy audio samples in the dataset, potentially caused by a malfunctioning recording device or proximity to an unidentified noise source. We conducted a preprocessing phase, which dealt with analyzing high-level audio features, extracted from the SiCRNN model. This phase involved overfitting the network with data from each single patient, and assessing the “separability” of the embedding vectors, belonging to the *positive* and *negative* classes. A visual evaluation was initially performed by using principal component analysis (PCA) to reduce the embeddings’ dimensionality and allow for two-dimensional plots. The scatter plots of Figure 1a,b show examples of noise-free and noisy embedding vectors after network overfitting. We also assessed the presence of corrupted samples by measuring the distances between the centroids of the *positive* and *negative* point clouds. For most patient data, the network was able to separate *apnea* and *non-apnea* events, achieving an average distance between class embeddings of 1.99±0.11. However, three patients reported a separability significantly below this range, (about 0.88), so they were excluded from the experiments.

Finally, the dataset, comprising the remaining 37 patients, was divided into three different data splits. Each data split contained a train, validation, and test partition: 30,000 items from 23 patients (about 65%) were used for training, 12,992 items from 6 patients (about 15%) were used for validation, and 15,162 items from 8 patients (about 20%) were reserved for the test. To ensure a fair evaluation, each patient was assigned exclusively to one partition.

Since *apnea* events were inherently less frequent than normal breathing episodes (only 30% of the total recording duration contained *apnea* events), we enforced the balancing of *positive* and *negative* pairs in the training and validation sets by implementing oversampling of the minority class (*apnea*).

### 2.2. SiCRNN Architecture

The proposed SiCRNN architecture was built on the convolutional-recurrent configuration used in [28,29], detailed as follows: the input features consisting of Mel or linear spectrograms were processed using convolutional operators, with a unitary stride and the *same* padding, to preserve the original size after convolution. Each convolutional layer was followed by batch normalization and rectified linear unit (ReLU) activation. Non-overlapping max-pooling was then applied to the feature map, reducing data dimensionality by compressing it in both the time and frequency domains. The SiCRNN consisted of a stack of convolutional blocks, which included convolution, normalization, pooling, and activation. The kernel size was kept the same while the pooling size increased.

After stacking the feature maps along the frequency axis, the outputs of the CNN backbone were fed into the bidirectional gated recurrent unit (GRU) layers. GRU selectively updates and uses information from previous time steps, allowing it to capture long-term dependencies in time series [30,31].

### 2.3. Training Strategy

**Figure 2:**
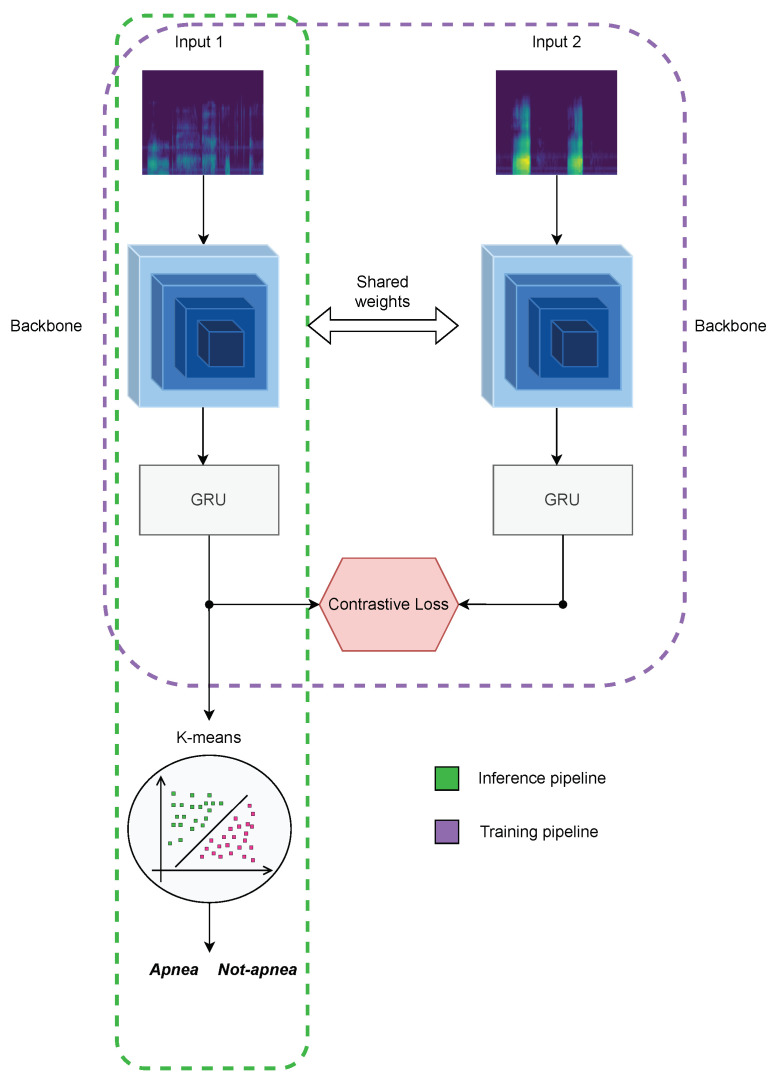
Overview of the proposed SiCRNN framework. The purple dashed line highlights the Siamese configuration employed during the training phase, whereas the green dashed line corresponds to the inference phase, which is carried out through the k-means clustering algorithm.

The training of the proposed framework was carried out in two stages. During the first training stage, a Siamese-like strategy was followed, feeding two identical neural network architectures with either positive (*apnea*–*apnea*) or negative (*apnea*–*non-apnea*) input pairs. This is depicted in Figure 2, where the two architectures shared weights during the two-step forward process, characteristic of Siamese networks [32]. The resulting 128-dimensional embedding vectors for each input pair were then compared with a contrastive loss, to minimize intra-class distances, pulling vectors representing the same class closer together, and maximizing inter-class distances, pushing vectors from different classes further apart. A more detailed description of the contrastive loss is reported in Section 3.1.

In the second training stage, the embedding vectors were processed with the k-means clustering algorithm [33]. The latter computes the centroids of the clusters of points, representing *apnea* and *non-apnea* events [34].

During the validation and test phases, 6-s frames from the audio recording were processed by the model, and the resulting embedding vectors were compared with the coordinates of the previously calculated centroids, to assign the label of *apnea* or *non-apnea* to the frame under test, according to the shortest Euclidean distance.

## 3. Experimental Protocol

### 3.1. Training Settings and Performance Metrics

The maximum available batch size of 128 was used, according to the specifications of the test lab workstation, which was equipped with an Nvidia Titan RTX GPU with 24 GB VRAM.

We used a contrastive loss function to minimize intra-class distances by bringing vectors of the same class closer together and to maximize inter-class distances by pushing vectors from different classes apart. The best model was selected based on the one that minimized the loss during the training phase.

The mathematical formulation of the loss function is reported below:

$$L_{c}=\frac{1}{N}\sum_{i=1}^{N}\left(\left(1-{\mathrm{label}}_{i}\right)\cdot e_{d}^{2}+{\mathrm{label}}_{i}\cdot {\left(\max(0,m-e_{d})\right)}^{2}\right)$$

where

- $L_{c}$ is the loss function.
- *N* is the number of instances in the dataset.
- ${\mathrm{label}}_{i}$ is the label of the *i*-th example.
- $e_{d}$ is the Euclidean distance between two instances in the dataset provided as input.
- *m* is a margin constant that serves to control how much the representations of samples from different classes need to be separated in the embedding space. For our purposes, *m* was experimentally set to 2.

The performance of our approach was assessed using Recall, Precision, and the *F*1 *score* [35]. Specifically, Recall focuses on a DL model’s ability to identify the true positives (TPs) within the dataset. This is relevant in clinical applications as the cost of false negatives (FNs) (i.e., not recognizing a clinical condition when it is present) is high.

(1) $$Recall=\frac{{TP}_{i}}{{TP}_{i}+{FN}_{i}}$$

where *i* stands for the *i*-th class (which, for our purposes, is the *apnea* class) and ${TP}_{i}$ denotes the correctly classified apnea sample. Conversely, ${FN}_{i}$ refers to those samples that belong to the *apnea* class but have been incorrectly classified as *non-apnea*.

On the other side, Precision is used to measure how often a model correctly predicts the positive class (*apnea*).

(2) $$Precision=\frac{{TP}_{i}}{{TP}_{i}+{FP}_{i}}$$

where false positive (${FP}_{i}$) refers to those samples that are predicted as the *apnea* class but are labeled as the *non-apnea* class.

Instead, the *F*1 *score* is a measure that combines precision and recall into a single metric, treating both metrics equally [36]. It ranges from 0 to 1, where 1 represents a perfect model that makes all correct predictions, and 0 indicates a model with no correct predictions. Essentially, a higher F1 score signifies better performance, as it reflects a balance between the ability to correctly identify positive instances (Recall) and the accuracy of those positive predictions (Precision).

(3) $$F1=2\cdot \frac{Precision\cdot Recall}{Precision+Recall}$$

### 3.2. SiCRNN Tuning

Hyperparameter tuning was performed, focusing on the key components of the SiCRNN architecture (see Table 1). Given a large number of possible parameter combinations, we employed an optimized search strategy based on the approach proposed in [37].

Additionally, a frequency sensitivity analysis was conducted. Observing that most acoustic energy, during apnea episodes, is concentrated in a low-frequency range of the MEL spectrogram, we evaluated the model’s performance using different lower-dimensional inputs. To that end, the MEL spectrogram input feature map was resized by discarding the highest frequency bands, obtaining five configurations (see Table 2) that were included in the hyperparameter search process, even though they are not strictly referred to network parameters. Furthermore, at the lowest frequency range, we considered the use of the STFT spectrogram without perceptual frequency filtering, in order to understand how the compression applied by the Mel filterbank affects the model’s performance [38]. In both the Mel-scaled and linear frequency spectrogram calculations, a window of 2048 samples was used, with a hop size of 256. Experiments revealed that there is no direct relationship between input size and classification performance, as will be outlined later.

The hyperparameter tuning phase was conducted by training the network on a subset of 10,000 instances out of the 30,000 in the original training set while using the validation and test set, as previously defined. This choice was dictated by practical considerations, due to the need to repeat several instances of the training process.

In the end, only the best hyperparameter combinations, chosen from those with the highest F1 score in the validation set for each type of input, were retrained using the full dataset as presented in Section 2.1, consisting of 30,000 instances.

### 3.3. State-of-the-Art Comparison

Our study included a comparative analysis with the DL model proposed by Ding et al. [25], which is the most recent and closely aligned with our work. This model employs a sound event detection (SED) approach, using a VGG19 [39] backbone for feature extraction, three LSTM layers for temporal characterization, and three dense output layers to distinguish between snoring events associated with *apnea* and those unrelated to *apnea*. Following the workflow in [25], the network was pre-trained on the ImageNet dataset and fine-tuned on our dataset. Hereinafter, we will refer to this model as VGG19+LSTM.

The VGG19+LSTM model encompasses an end-to-end architecture, where input generation involves randomly extracting 6-s windows from the dataset, which are then individually passed through the model. Additionally, the number of elements in each class during the training phase is balanced to ensure equal representation.

In this case as well, to perform a comprehensive and detailed comparative analysis, tuning of the input features passed to the network was carried out by using the hyperparameter dataset, as described at the end of Section 2.1. However, it was not possible to perform a complete hyperparameter tuning on VGG19, as was done with the SiCRNN, because the former adopts a predefined configuration. Additionally, since we replicated the work conducted by other researchers, we decided to maintain the hyperparameter configuration proposed in the reference paper except for the types of features in the model input.

## 4. Results

**Figure 3:**
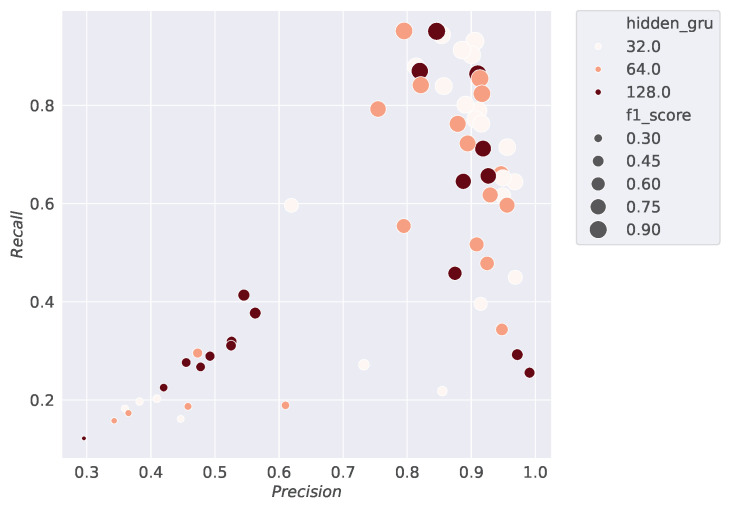
The scatter density plot shows the results of the hyperparameter tuning by relating the Precision, Recall, and *F*1 *score* metrics to the number of GRU hidden layers used during training. On the x-axis, Precision values are reported, while the y-axis represents Recall values, and the size of the points indicates the *F*1 *score*. The different shades of orange represent the number of convolutional blocks used in the model’s training.

**Figure 4:**
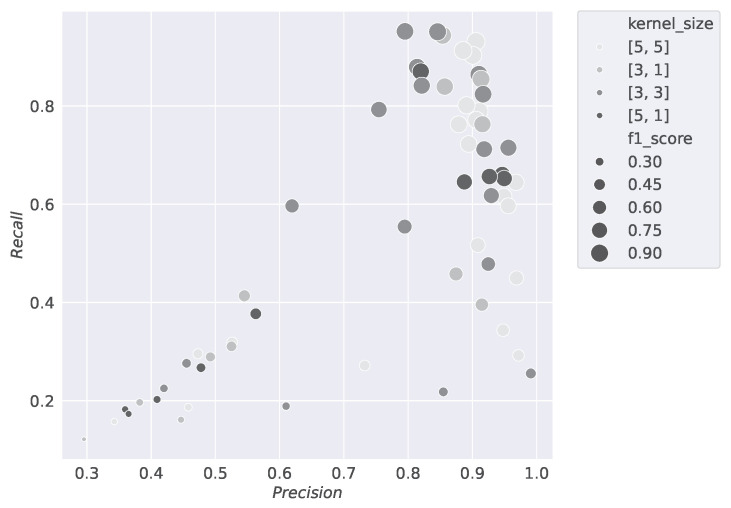
The scatter density plot shows the results of the hyperparameter tuning by relating the Precision, Recall, and *F*1 *score* metrics to the dimension of the kernel size used during training. On the x-axis, Precision values are reported, while the y-axis represents Recall values, and the size of the points indicates the *F*1 *score*. The different shades of gray represent the kernel size used in the model’s training.

**Figure 5:**
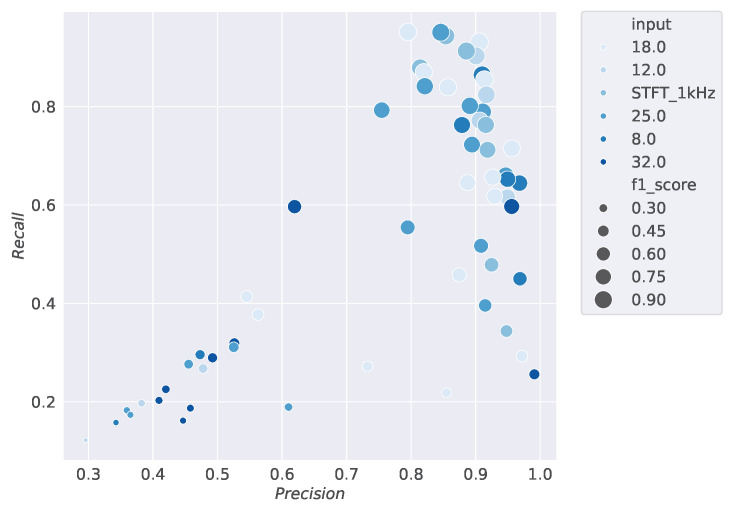
The scatter density plot shows the results of the hyperparameter tuning by relating the Precision, Recall, and *F*1 *score* metrics to the number of MEL bands selected for each input sample frequency during training. On the x-axis, Precision values are reported, while the y-axis represents Recall values, and the size of the points indicates the *F*1 *score*. The different shades of blue represent the number of MEL bands used in the model’s training.

**Figure 6:**
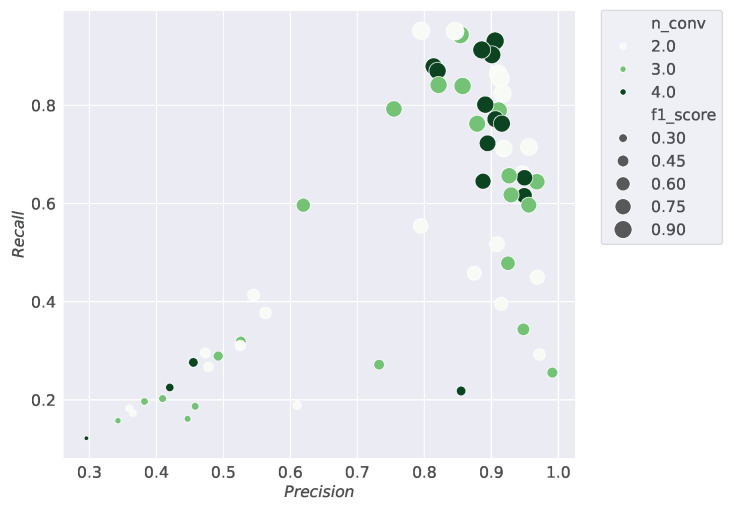
The scatter density plot shows the results of the hyperparameter tuning by relating the Precision, Recall, and *F*1 *score* metrics to the number of convolutional blocks used during training. On the x-axis, Precision values are reported, while the y-axis represents Recall values, and the size of the points indicates the *F*1 *score*. The different shades of green represent the number of convolutional blocks used in the model’s training.

This section displays the results of the conducted studies. Figure 3, Figure 4, Figure 5 and Figure 6 show the results of the hyperparameter tuning for the SiCRNN model, performed using a random search approach on 60 random combinations of the hyperparameters described in Section 3.2.

Specifically, Figure 3 highlights how the model’s performance is influenced by the number of hidden layers in the GRU module; Figure 4 shows how the metrics vary with changes in the convolutional kernel size; Figure 5 relates the metrics to input dimensionality, while Figure 6 illustrates the impact of the number of convolutional blocks on the metrics.

Looking at Figure 3, Figure 4, Figure 5 and Figure 6, the larger dots positioned toward the upper right corner indicate the configurations that achieve the best performance among those tested during hyperparameter tuning, demonstrating how the selection of the right hyperparameters can significantly influence the model’s performance.

The results of the optimal configurations for each type of input are presented in Table 3, while Table 4 shows the results obtained by the VGG19+LSTM model, for the same input types.

Table 5 below lists the resources required by the two models under comparison, in terms of trainable parameters, floating points operations (FLOPS) in forward time, input memory size, and total network memory footprint.

We carried out additional experiments to compare the performance of the proposed SiCRNN model with that of VGG19+LSTM. Using the random hold-out method, we generated multiple random data splits and conducted three separate tests, each using distinct training, validation, and test sets while adhering to the patient-level split procedure. The results of this validation are presented in Table 6; as shown, the proposed method outperforms the state-of-the-art, demonstrating robustness to data variability and achieving high average values with low variability in the results.

It is also important to highlight that the adoption of the k-means clustering algorithm in this study is underpinned by two primary considerations following the work in [40]. First, its suitability for distance-based Siamese neural networks makes it a compelling choice. These networks transform features extracted from input data into a representation space where similar elements are spatially proximate, forming well-defined clusters of points. This intrinsic organization makes k-means effective for classifying the embeddings generated by the Siamese backbone. The second consideration deals with computational efficiency: indeed, the k-means algorithm has a lower computational burden. This characteristic aligns well with the objectives of this research, which seeks to propose an effective methodology that can be seamlessly deployed in real-world applications, particularly those constrained by limited computational resources. Table 7 provides evidence supporting the advantages of k-means in terms of both performance and inference time. Specifically, the k-means algorithm achieves a superior *F*1 *score*, Precision, and Recall compared to support vector classifiers (SVCs) and k-nearest neighbors (KNN). Additionally, its inference time of 0.09 s is faster than those of SVC (42.33 s) and KNN (0.37 s). The results and considerations presented in the following sections pertain to the use of the k-means algorithm, as it proves superior to the other read-out methods.

For the correct reproduction of the results, we present in Table 8 the IDs of the patients used in each data split. It is also possible to find the codes used in the GitHub repository https://github.com/LilloByte/SiCRNN.git.

### Qualitative Results

**Figure 7:**
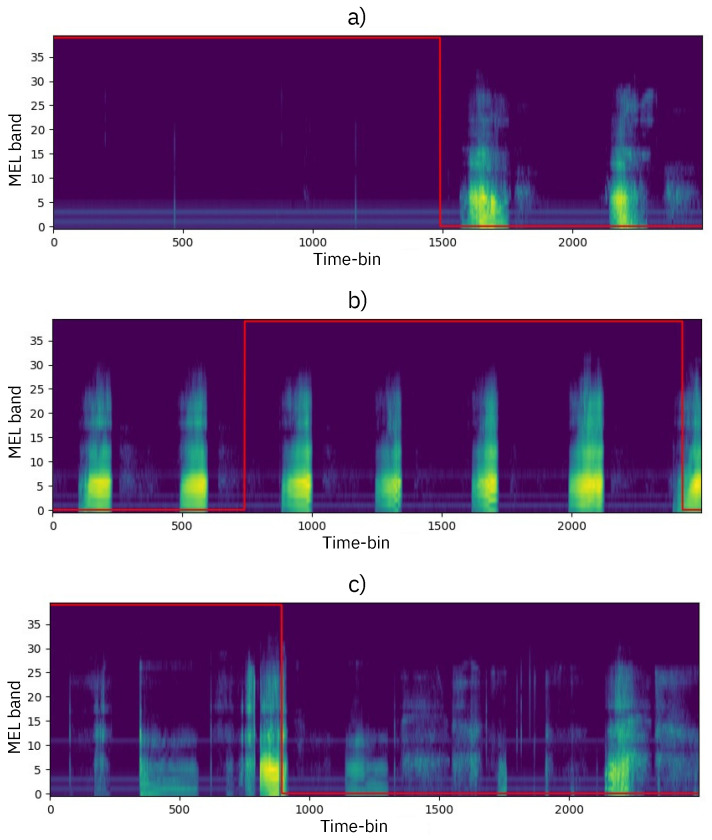
(**a**) The region located below the top of the red mask indicates the apnea events; (**b**,**c**) spectrograms with the labeled red mask display an apnea event with significant spectral content. The time associated with each individual bin in the spectrograms is 11.56 ms.

Upon conducting a qualitative analysis of the dataset, we found that identifying *apnea* events is not always straightforward. For example, in Figure 7a, the *apnea* event (marked by the top section of the red mask) can be visually recognized due to the absence of acoustic energy. Once the *apnea* event ends, breathing resumes and is evident in the spectrogram as periodic bursts of energy, representing inhalation and exhalation sounds.

In contrast, Figure 7b,c depict cases where the *apnea* event is indistinguishable from normal breathing, as the spectrogram shows significant energy within the segment, labeled as *apnea* by experts, making machine detection more difficult.

Spectrograms with patterns similar to those in Figure 7a are correctly classified by the proposed method, whereas instances like Figure 7b or Figure 7c may lead to false negatives during the network’s prediction stage.

## 5. Findings and Discussion

In the following, we discuss the data reported in Section 4. The experimental results of the SiCRNN hyperparameter tuning (Table 3) show how performance is affected by parameters such as the input type, Mel bands, convolutional kernels, convolutional layers, and recurrent layers. Performance metrics such as the *F*1 *score*, Recall, and Precision are analyzed, along with computational parameters, such as the number of training parameters, FLOPS, and input size, which are reported in the upper section of Table 5.

Concerning the sampling rate (SR) of the audio input, both 1 kHz Mel and STFT spectrograms exhibit strong performance. Specifically, the Mel configuration with 8 bands and a convolutional kernel size of [3,3] achieves an *F*1 *score* of 0.89, with a Recall of 0.86 and a Precision of 0.91, indicating a good balance between Recall and Precision. Similarly, the STFT configuration, which uses a larger kernel [5,5] and 4 convolutional layers, maintains a similar *F*1 *score* (0.89) but shows an improvement in Recall (0.93), suggesting a higher capacity to detect events, although Precision remains stable at 0.86. However, this configuration incurs a significantly higher computational cost, with FLOPS reaching 0.13 G and a total model size of 41.06 MB, compared to 7.65 MB for the Mel configuration. This suggests that Mel provides a more efficient trade-off between performance and computational complexity.

With a 2 kHz SR, performance improvements are observed with the Mel input and 12 bands. The *F*1 *score* reaches 0.9, with a very high Recall of 0.95 and stable Precision at 0.86. However, computational complexity increases significantly compared to the 1 kHz Mel configurations, with FLOPS at 0.33 G and a model size of 13.07 MB.

Configurations with higher SR, such as 4, 8, and 16 kHz, maintain high performance, with the *F*1 *score* hovering around 0.88–0.89. However, slight declines in Recall and Precision are noted, especially at 8 kHz, where the *F*1 *score* drops to 0.85 and Recall to 0.79, while Precision remains high at 0.93. This trend suggests that increasing the SR and Mel bands does not necessarily lead to a linear improvement in performance, and there may be an optimal point, as indicated by the results at 2 kHz.

The number of hidden units in the GRU layer varies between 32, 64, and 128, but there does not seem to be a direct correlation between the number of hidden units and model performance. For example, configurations with 128 units perform well, but even configurations with only 32 units compete in terms of F1, Recall, and Precision.

In conclusion, the results suggest that the 2 kHz configuration with the Mel input and 12 bands represents the best trade-off between performance and computational complexity, offering an excellent balance of Recall, Precision, and F1. Higher frequency configurations, while delivering good results, involve a significant increase in required resources without substantial improvement in performance metrics. Lower frequency configurations, such as 1 kHz and 8 Mel bands, remain competitive, especially in contexts with computational efficiency constraints.

Table 4 and Table 5 allow us to compare the performance and computational cost of the VGG19+LSTM model for different inputs. It is worth noting that the SR, which ranges from 1 kHz to 16 kHz, has an adverse impact on the model’s performance. The 1 kHz configuration with the Mel input and 8 bands achieves the highest *F*1 *score* (0.75). Its Recall and Precision are 0.84 and 0.68, respectively. The low SR configuration allows the VGG19+LSTM to detect events sufficiently well but it suffers from a high number of false positives.

When STFT is used instead of Mel with the same 1 kHz SR, the model displays a slightly lower performance, with an *F*1 *score* of 0.71. Although Recall increases to 0.85, Precision drops to 0.61, indicating that STFT improves the model’s ability to correctly identify events but with reduced Precision. Additionally, the STFT configuration requires significantly more computational resources; FLOPS increase to 9.19 G, and the total model size grows to 195.21 MB, making this configuration much more computationally expensive compared to Mel.

Increasing the SR to 16 kHz with the Mel input and 32 bands leads to a slightly lower *F*1 *score* of 0.70. This means that, despite the increased spectral resolution, there is no significant improvement in the overall model performance despite an increase in computational complexity. At 16 kHz, FLOPS reach 4.6 G, and the total model size rises to 145.62 MB.

Examining intermediate configurations with SRs of 2, 4, and 8 kHz, the *F*1 *score* remains relatively stable at around 0.71, with a slight decrease to 0.66 at 8 kHz. Precision and Recall follow a similar trend, indicating that increasing the SR does not lead to a significant improvement in performance. However, the increase in SR results in growing computational demands, with FLOPS rising from 1.6 G to 3.56 G and the total model size increasing from 111.25 MB to 133.13 MB.

Overall, the experimental results of the VGG19+LSTM method indicate that the 1 kHz configuration with 8 Mel bands offers the best trade-off between performance and computational costs, but the *F*1 *score* is much lower than the one obtained by the proposed method.

The results show that an increase in input dimensionality does not positively impact the performance of the SiCRNN model. On the other hand, changes in input dimensionality affect the computational cost of the model and memory usage In real clinical settings, large computational resources are not always guaranteed, making it preferable to have lightweight models. With our approach, we can ensure higher performance than VGG19+LSTM while significantly reducing the computational load.

The strong performance of the SiCRNN can be credited to the effectiveness of the Siamese methodology [41], widely applied in anomalous sound detection [42] or in data scarcity scenarios [43]. This technique excels in enhancing the separation between two event categories more efficiently than conventional binary classification models. Additionally, Siamese networks take advantage of a potentially extensive set of training data pairs, enabling the model to make effective use of even a small number of instances from the minority class, such as *apnea* events, as demonstrated in the work by Schroff et al. [44].

## 6. Conclusions

This study introduces a novel clinical decision support system designed to identify SAS events using audio signals recorded from tracheal microphones. Although PSG is the gold standard for diagnosing SAS, it is invasive and heavily reliant on subjective clinical assessments. To address these challenges, we propose a deep learning-based approach that employs a SiCRNN in combination with k-means clustering for effective *apnea* event detection.

The proposed SiCRNN model, trained on Mel audio spectrograms, exhibited a strong ability to distinguish between *apnea* and *non-apnea* events, surpassing other state-of-the-art methods. This model takes advantage of the Siamese training paradigm, which enhances the distinction between *apnea* and *non-apnea* events, making it highly suitable for SAS detection. This study uses a subset of the Korompili et al. dataset, ensuring high-quality data by filtering out corrupted recordings during preprocessing.

Experimental results highlight the effectiveness of the SiCRNN model compared with another deep learning model, VGG19+LSTM, underscoring the superior performance of the SiCRNN despite its fewer trainable parameters. This success can be attributed to the model’s efficient feature extraction and the Siamese approach’s ability to make optimal use of limited minority class examples of *apnea* events.

In conclusion, the SiCRNN framework marks a significant step forward in the non-invasive identification of SAS, offering a promising alternative to PSG by enabling accurate, automated *apnea* detection through tracheal audio analysis. This method shows potential for widespread clinical application, particularly in home settings, which could facilitate early diagnosis and management of sleep apnea.

Future directions for this project will focus on improving the performance of the decision support system by incorporating additional post-processing techniques to refine classifier output and introducing more clinically oriented metrics, such as the Apnea-Hypopnea Index (AHI), for objective assessment [45,46].

## Figures and Tables

**Table 1:** Hyperparameter search space.

Parameter	Values
CNN kernel size	[5,5], [3,3], [5,1], [3,1]
Convolutional blocks	2, 3, 4
GRU hidden layers	32, 64, 128

**Table 2:** Different types of inputs considered for the frequency sensitivity analysis.

Input Type	MEL Bands	Frequency Range [Hz]	Input Size
MEL	8	0–500	[8,374]
MEL	12	0–1000	[12,374]
MEL	18	0–2000	[18,374]
MEL	25	0–4000	[25,374]
MEL	32	0–8000	[32,374]
STFT	-	0–500	[1025,24]

**Table 3:** Results obtained for each type of input, in the best configuration of hyperparameters, after being retrained on the entire dataset. The highest values are shown in bold.

Input Type	MEL Bands	Kernel Size	Conv. Layers	Hidden GRU	*F1*	*Recall*	*Precision*
MEL	8	[3,3]	2	128	0.89	0.86	0.91
MEL	12	[5,5]	4	32	**0.90**	**0.95**	0.86
MEL	18	[5,5]	4	32	0.89	0.91	0.88
MEL	25	[3,3]	2	128	0.85	0.79	**0.93**
MEL	32	[5,5]	3	64	0.88	0.87	0.90
STFT	-	[5,5]	4	32	0.89	0.93	0.86

**Table 4:** Results of the best configurations for each input type of the VGG19+LSTM model after being retrained on the entire available dataset. The highest values are shown in bold.

Input Type	MEL Bands	*F1*	*Recall*	*Precision*
MEL	8	**0.75**	0.84	**0.68**
MEL	12	0.71	0.79	0.65
MEL	18	0.71	0.81	0.64
MEL	25	0.66	0.77	0.58
MEL	32	0.70	0.72	**0.68**
STFT	-	0.71	**0.85**	0.61

**Table 5:** Resource consumption and memory impact for the proposed SiCRNN and the VGG19+LSTM architectures.

Model	Input Type	MEL Bands	Params (M)	FLOPS (G)	Input Size (MB)	Total Size (MB)
SiCRNN	MEL	8	0.60	0.04	0.01	7.65
	MEL	12	1.1	0.33	0.02	13.07
	MEL	18	1.1	0.73	0.03	18.67
	MEL	25	0.60	0.11	0.04	18.14
	MEL	32	0.86	0.33	0.05	22.87
	STFT	-	1.1	0.13	0.09	41.06
VGG19+LSTM	MEL	8	24	1.27	0.01	105.37
	MEL	12	24	1.60	0.02	111.25
	MEL	18	24	2.39	0.03	120.81
	MEL	25	24	3.56	0.04	133.13
	MEL	32	24	4.60	0.05	145.62
	STFT	-	24	9.19	0.09	195.21

**Table 6:** Comparison of the F1 score (F1), Recall and Precision across different data splits (Split 1, Split 2, Split 3) for the proposed SiCRNN and the comparative method VGG19+LSTM. The results are presented in term of mean value and standard deviation (in brackets).

	SiCRNN			VGG19+LSTM		
	*F1*	*Recall*	*Precision*	*F1*	*Recall*	*Precision*
Split 1	0.90	0.90	0.90	0.75	0.84	0.68
Split 2	0.91	0.91	0.92	0.62	0.78	0.51
Split 3	0.90	0.88	0.92	0.64	0.91	0.50
Mean (SD)	**0.90 (0.008)**	**0.90 (0.01)**	**0.91 (0.01)**	0.67 (0.07)	0.84 (0.06)	0.56 (0.1)

**Table 7:** Performance comparison between k-means, support vector classifier (SVC), and k-nearest neighbors (KNN) read-out algorithms. The results are presented in terms of the mean value and standard deviation (in brackets) of Precision, Recall, and *F*1 *score* (F1) metrics.

Read-Out	*F1*	*Precision*	*Recall*	Inference Time (s)
K-means	** 0.90(0.008) **	** 0.90(0.01) **	** 0.91(0.01) **	** 0.09 **
SVC	0.73(0.01)	0.72(0.03)	0.73(0.04)	42.33
KNN	0.73(0.01)	0.75(0.02)	0.70(0.03)	0.37

**Table 8:** Summary of patient IDs used for each data split divided into training, validation, and test sets.

	Train	Validation	Test
**Split 1**	1112, 1110, 1108, 1106, 1095, 1093, 1089, 1088, 1086, 1082, 1071, 1069, 1057, 1045, 1041, 1039, 1037, 1028, 1022, 1010, 1008, 1006, 995	1120, 1104, 1043, 1024, 1018, 1014	1118, 1116, 1073, 1059, 1026, 1020, 1000, 999
**Split 2**	1120, 1118, 1116, 1112, 1110, 1108, 1106, 1104, 1095, 1093, 1089, 1088, 1086, 1082, 1073, 1071, 1069, 1059, 1057, 1045, 1043, 1041, 1039	1010, 1008, 1006, 1000, 999, 995	1037, 1028, 1026, 1024, 1022, 1020, 1018, 1014
**Split 3**	1086, 1082, 1073, 1071, 1069, 1059, 1057, 1045, 1043, 1041, 1039, 1037, 1028, 1026, 1024, 1022, 1020, 1018, 1014, 1010, 1008, 1006, 1000	1120, 1118, 1116 1112, 999, 995	1110, 1108, 1106, 1104, 1095, 1093, 1089, 1088

## Data Availability

The materials needed to reproduce the experiments and results of this study are publicly available in the GitHub repository at https://github.com/LilloByte/SiCRNN.git. No new data were generated in this study.
